# Patients' and Practitioners' Views of Knee Osteoarthritis and Its Management: A Qualitative Interview Study

**DOI:** 10.1371/journal.pone.0019634

**Published:** 2011-05-05

**Authors:** Sophie Alami, Isabelle Boutron, Dominique Desjeux, Monique Hirschhorn, Gwendoline Meric, François Rannou, Serge Poiraudeau

**Affiliations:** 1 Interlis, Université Paris Descartes, Paris, France; 2 AP-HP, Centre d'Epidémiologie Clinique, Université Paris Descartes, Paris, France; 3 Université Paris Descartes, Paris, France; 4 Pfizer, Paris, France; 5 AP-HP, Service de Rééducation et Réadaptation de l'Appareil Locomoteur et des Pathologies du Rachis, Université Paris Descartes, INSERM IFR 25 Handicap, Paris, France; Marienhospital Herne - University of Bochum, Germany

## Abstract

**Purpose:**

To identify the views of patients and care providers regarding the management of knee osteoarthritis (OA) and to reveal potential obstacles to improving health care strategies.

**Methods:**

We performed a qualitative study based on semi-structured interviews of a stratified sample of 81 patients (59 women) and 29 practitioners (8 women, 11 general practitioners [GPs], 6 rheumatologists, 4 orthopedic surgeons, and 8 [4 GPs] delivering alternative medicine).

**Results:**

Two main domains of patient views were identified: one about the patient–physician relationship and the other about treatments. Patients feel that their complaints are not taken seriously. They also feel that practitioners act as technicians, paying more attention to the knee than to the individual, and they consider that not enough time is spent on information and counseling. They have negative perceptions of drugs and a feeling of medical uncertainty about OA, which leads to less compliance with treatment and a switch to alternative medicine. Patients believe that knee OA is an inevitable illness associated with age, that not much can be done to modify its evolution, that treatments are of little help, and that practitioners have not much to propose. They express unrealistic fears about the impact of knee OA on daily and social life. Practitioners' views differ from those of patients. Physicians emphasize the difficulty in elaborating treatment strategies and the need for a tool to help in treatment choice.

**Conclusions:**

This qualitative study suggests several ways to improve the patient–practitioner relationship and the efficacy of treatment strategies, by increasing their acceptability and compliance. Providing adapted and formalized information to patients, adopting more global assessment and therapeutic approaches, and dealing more accurately with patients' paradoxal representation of drug therapy are main factors of improvement that should be addressed.

## Introduction

Society must prepare itself for an aging world. Arthritis (mainly osteoarthritis [OA]) is the most common cause of reported disabilities [1], [2]. Hence, disability and participation restriction is becoming an important component to assess in defining public health strategies.

The patient point of view regarding health status has gained importance in decision-making procedures and has been considered a possible criterion standard to assess treatment efficacy [3]. Results of a recent French survey suggest that the burden of knee OA in primary care is substantial [4], and a substantial decrease in health-related quality of life (HRQoL) was also reported in a family practice setting [5], [6]. However, disability and HRQoL are usually measured by fixed-item questionnaires that do not take into account patient priorities. A survey conducted in primary care suggested that patients perceived knee OA to be more disabling than hypertension, diabetes mellitus and heart diseases, whereas physicians considered these 3 latter conditions the most important chronic conditions [7]. Patients with knee and hip OA or rheumatoid arthritis (RA), healthy professionals, and healthy controls do not agree on the importance of disabilities [8], [9]. These discrepancies between patients and physicians in defining the importance of an illness associated with substantial decreases in HRQoL should lead to a paradigmal shift toward a more patient-centred approach. Taking into account patient priorities may lead to a better understanding of what is important to them [10].

Although patients with OA and their physicians may differ in their assessment of what is important in health and symptom status [11], views of patients and practitioners concerning knee OA management have been seldom studied. A qualitative study involving semi-structured interviews of German patients with OA, nurses, and general practitioners (GPs) suggested that GPs should focus more on disability and pain and on giving information about treatment [12]. Qualitative research is probably the best way to understand patients' needs and contexts and could improve therapeutic strategies and their assessment [13]. The US Food and Drug Administration has recently proposed guidelines for patient-reported outcomes that emphasize the need for semi-structured interviews of patients to ensure content validity of these instruments [14]. We aimed to qualitatively assess patients' and physicians' views concerning knee OA and its management by using semi-structured interviews.

## Methods

### Ethics statement

All patients gave their written informed consent to participate in the study. The study protocol was approved by the ethics committee of Cochin Hospital, Paris. Investigations were conducted according to the principles of the Declaration of Helsinki.

### Qualitative interview study

This was a qualitative interview study of patients and care providers conducted according to guidelines for inductive qualitative research [15], [16].

Semi-structured interviews were conducted with both patients and care providers to explore patients' and care providers' views about knee OA management. Individual behaviours (attitudes and practices), personal feelings and interpretations, social interactions and material backgrounds were specifically examined throughout the patients' therapeutic journey, to allow for a deep understanding of patients' expectations and fears and beliefs and practitioners' expectations.

### Sample

A heterogeneous sample of 81 patients and 29 care providers was selected. The sample selection was based on non-probability judgment sampling, assuring both relevance to the subject and diversity of the members selected [17]. The diversity of the care providers' sample was ensured for age (<45 years, n = 11), gender (8 women), specialty (11 GPs, 6 rheumatologists, 4 orthopedic surgeons, 8 [4 GPs] delivering alternative medicine), and place of practice (23 urban/6 rural). The diversity of the patient sample was ensured for age (45–60 years, n = 29; 61–80 years, n = 38; >80 years, n = 14), gender (59 women), professional activities (yes, n = 34; retired, n = 57), and place of living (55 urban/6 rural). This quite large sample size for a qualitative study is explained by the limited data available on the subject, the diversity of the population concerned by knee OA and the exploratory nature of the research. The patients were selected from files of care providers not involved in the interview process.

### Interviews

After a study of the literature on evidence-based procedures and guidelines for knee OA, interviews of experts in the field and patients' perspectives of chronic diseases, we compiled semi-structured interview guides with open-ended questions. Interview guides for both groups were as similar as possible to allow comparison across groups.

The interview guides were structured by combining a “funnel-shaped” structure and the “itinerary method” [18]–[22]. The funnel-shaped structure was adopted to ensure that the interviews allowed for an inductive comprehension of the social reality at stake beneath the knee OA situation. The itinerary method of data collection was derived from anthropological data collection techniques and focused on objects, practices and the decision-making process. Applied to a therapeutic situation, the method allows the researcher to follow the course of the situation for the patient, from the appearance of the abnormality to the time of the interview, thus placing knee OA in a broader context than the medical one. The postulate underneath this framework is that patients' views on knee OA management cannot be limited to the collection of explicit expectations that the patients can possibly express: they have to be identified throughout an analysis of the global social situation in which knee OA occurred and was (or was not) managed, identifying contradictions, ambivalence, implicit expectations or unanswered needs.

The interview guides thus combined a thematic structure (views of OA, its effects and the following adjustments, description and evaluation of the patients' therapeutic journey, expectations, and fears and beliefs) with chronological sequences to detail the therapeutic journey and the course of consultation: diagnostic routines, information giving, prescribing, advice for lifestyle, and referrals. For physicians, the interview guide covered practitioners' views of arthritis and knee OA (specificity, causes, limitations and social impacts, evolution); the description of the management of knee OA to analyze decision-making processes (different sequences were detailed, such as the diagnosis process[es] and routine[s]) [interrogatory, physical examination, announcement of the diagnosis, counseling, etc.]; and therapeutic decision-making processes [including renewal, adjustment and modification of prescriptions, referral to another physician, uncertainties encountered], the description of the patient–practitioner interactions at all steps of the therapeutic journey (identifying questions asked, information delivered, subjects discussed, patients' resistance or specific demands, and social strategies adopted), and practitioners' expectations.

The mean time for these interviews was 1.5 hr for patients and 1 hr for physicians Interviews for 8 patients were structured as “life history interviews” focusing on knee OA and lasted 2 hr. We used the life history technique to question the social construction of the views of knee OA and its management. Classically, we proceeded chronologically, asking the interviewees to describe their childhood right up to the present day. Nevertheless, we adapted this technique by focusing on the life story with arthritis and its management to look at family stories and identify opinions, behaviour or attitudes related to arthritis that might have been passed down through generations. In the case of arthritis, family stories are used to interpret personal experiences, for self-diagnosis, and to evaluate the gravity of the illness and “heredity”. The stories appeared to work as a reinforcement of medical diagnosis. In the specific case of arthritis, life history interviews revealed confusion between “rheumatism” and “arthritis” in the patients' minds.

### Procedures

All patients but 14 who preferred public places were interviewed at home by trained interviewers. Care provider interviews took place at practice locations. During the interview, the interviewer ensured that every aspect was explained sufficiently and in detail.

### Analysis

The conversations were recorded digitally, transcribed literally and analyzed by 4 researchers (all sociologists). An initial categorizing system was established on the basis of the interview guides. This first thematic index was modified, categories and subcategories being added as they emerged from the analysis of the data and researchers continually checking that they had a common understanding of the categories generated. Numerous free categories were developed, discussed, adjusted and grouped in an iterative and inductive process. All data were coded according to the final thematic index generated.

## Results

### Patient views

Two main domains were identified: one about the patient–physician relationship and the other about treatment.

### Patient–physician relationship

#### Sources of satisfaction

Confidence with the practitioner seemed to determine the relationship and depend on a combination of factors. One factor was the feeling of being in a specific and individualized relationship with the care provider that gives the feeling that the physician is “their” doctor. This feeling was related to the interpersonal and communication skills of physicians and their ability to adopt a holistic approach to the patient:

*“This doctor, he doesn't know my case. When he comes home, he doesn't chat, he doesn't ask questions. Whereas the other doctor (the one I prefer), asks questions about my family, about my home. He is lovely. Sometimes, he waves at me while driving. The other one goes by as if he doesn't know me.” (Patient)*

*“Sometimes, there are patients that make mistakes but my doctor, he sorts out everything! He is really competent. He is kind and he has a real sense of humor. I do appreciate him a lot, because he is really human.” (Patient)*

This feeling also stemmed from specific behavior that conveyed the accessibility of the physician and ethical qualities such as devotion, conviction, prioritizing therapeutic over financial considerations, and resoluteness in disease management.

*“One day, for instance, I was in holiday with my husband. My knee was painful but I was out of anti-inflammatory. They didn't want to give me my treatment at the drug store as I had no prescription. I called my GP: he made me one, and faxed it to the drugstore. I am really pleased with him.” (Patient)*

*“I also have a GP I see very often. I can have an appointment in two days with him if I need one.” (Patient)*

*“Young doctors don't really care nowadays. Money is more important that humanity sometimes.” (Patient)*

Medical competence was also reported and estimated by the physician's estimated reputation, age and training. All these factors conveyed a sense of security to the patients, which is, to a certain extent, a way to deal with the uncertainty of their medical situation: uncertainty about the origin of the illness, the efficiency of treatment, and the evolution of the disease and its impact on their daily life. Moreover, this trusting relationship appeared to allow for patient cooperation and participation and for patients to be part of the medical decision-making process:

*“The doctor talks decently to you. He respects your identity, your wishes. He told me: I can operate now but if you want, I can also delay the surgery. It pleased me that he considered what I wanted.” (Patient)*

#### Sources of dissatisfaction

Sources of dissatisfaction were not totally compatible with sources of satisfaction. A main source of dissatisfaction was the physician accentuating the patient's feeling of uncertainty about OA by the patient feeling that they received unclear explanations or insufficient knowledge:

*“You know, doctors don't talk a lot. And I don't follow their jargon; I don't really understand what they say. They don't try (to be understood); they don't lose their time.” (Patient)*

*“I've never had answers to my expectations and to my questions before (I met this new doctor). I am interested in whatever information I can have because we (patients) are sorely in need of information.” (Patient)*

Practitioners trivializing OA and having fatalistic attitudes gave patients the feeling that their complaints were not recognized:

*“Anyway, I've always been skeptical about the knowledge and the interest of the care providers for osteoarthritis. They always gave me vague information, they are not able to precise the evolution of osteoarthritis. They are fatalists: they say that osteoarthritis is normal and that there is nothing to do. It shows clearly that physicians have a fatalistic attitude towards osteoarthritis that they are not concerned, not informed.” (Patient)*

Physicians imparting the feeling that therapeutic options are only palliative led patients to question the efficacy of what they call “modern medicine” for OA:

*“Classical medicine acts on symptoms. In other words, it decreases pain but it does not cure the cause.” (Patient)*

*“- It's the same when I have a headache, I take a painkiller … my headache calms down, but this does not solve the real problem… That's how I see it now (Patient)*

The systematic rejection by some physicians of alternative medicine options was also a source of dissatisfaction:

*“What I don't like is that doctors, whatever their speciality, do not recognize that there are alternatives to traditional medicine. They don't want to admit alternative medicine can also be efficient.” (Patient)*

#### Evolution of satisfaction

Satisfaction and dissatisfaction are not stable but are contingent and dynamic processes. Satisfaction with a practitioner results from complex processes mobilizing social, material, symbolic, and psychological factors, as well as priorities, which vary among individuals. These variations depend on OA evolution, the effect of health status on patients' lives, and patients' psychological status, and they evolve over time. Dissatisfaction does not seem to result from one cause but rather occurs with the progressive accumulation of factors leading to discontentment and finally to the rejection of treatments and sometimes practitioners. This process can lead to the disruption of the therapeutic relationship. Punctual dissatisfaction in a confident global relationship does not call into question this relationship and does not lead to disruption of this relationship. Finally, a long relationship does not necessarily mean satisfaction with the relationship:

*“- I have my current GP for 2 years now, and I had a family GP before. He was my GP for a very long time. But the thing is he took things lightly, so I stopped seeing him.*

*- (Can you tell me more about that?)*

*- He entered into the room; he took my pulse, listened, and nothing else.*

*- (What was he light about?)*

*- Well, he never changed his views: he was not really active. He did not make me do any blood tests, X-rays… If I wanted one, I had to ask: “Can we do an X-ray?” “Oh yes if you wish so” and so we did it. To me it is not a doctor…*

*- (How long did you see him?)*

*- 10 years at least.*

*- (Why did you wait so long to switch?)*

*- Well, I am not keen on changes… Not keen at all… I don't like to change my GP for another one, I can't deal with it … I am loathed of it…*

*- (Why?)*

*- I don't know! I don't know, but… one day I had an operation. I was sent to hospital and he never asked about me, nothing…At that time I felt offended (and then I switched for another GP).*

#### Treatments

Patients' views of treatments are various and ambivalent, which sheds light on an overall reserved appreciation of biomedical treatments for knee OA. Pharmacological treatments are considered useful for symptoms (immediate relief of pain) but unsuccessful for disease evolution. Patients' views of treatments differ depending on whether knee OA is considered an occasional or a chronic problem. Expectations of those considering knee OA an occasional problem are mainly symptom relief, whereas expectations of those considering knee OA a chronic problem are to dispose of a treatment being able to modify or stop OA evolution.

Patients' views of drug therapies for knee OA are paradoxical, the drugs being considered both therapeutic and noxious. This view generates fear and avoidance about drug therapies and a general attitude that could be designated “the less drug therapy possible”:

*“When I'm in the middle of big crisis, when I actually can't move, I take anti-inflammatory drugs but I am trying to avoid taking them as long as possible. I really have to be stuck for several days to take it.” (Patient)*

Patients' categorization of treatments for knee OA does not fully correspond to those of care providers. Complementary exams, especially imaging, are considered part of the treatments and are appreciated and awaited.

*Oral medications:* Analgesics are considered periodic symptomatic helpers. Their use is considered occasional, to anticipate a painful situation or to attenuate an existing symptom. Fear of side effects and dependency are reservations formulated by patients especially for opioids:

*“I became addictive to morphine, and I find this medicine really dangerous because… you don't feel the pain anymore. It's the ideal treatment for the pain, but it's a fake. To me it's a fake. Because when you meet professors, you deny you're suffering in a way, and when the professor asked me: “do you feel the pain madam?” How could I give a logical answer? You can only answer “no” because you don't feel anything anymore. And the more you take these pills, the more relieved you are, but it still is drugs…” (Patient)*

This view leads to dosage restriction for long-duration prescriptions.

Non-steroidal anti-inflammatory drugs (NSAIDs) are perceived as having an important risk of significant side effects:

*“I am against it, they are rubbish. All of them have side effects. The liver has to eliminate everything. That's why I was not keen on anti-inflammatory drugs.” (Patient)*

Their use is considered a periodic solution without regular renewal. Patients taking NSAIDs commonly reported their limited efficacy and the absence of long-term effects and expressed fears about tolerance with regard to limited treatment options.

*“It's also for this reason I don't want to take too much medicine. I am scared it will not do anything otherwise, especially when I will need it, later. (…)I do not to take too much because I know it can create addiction issues. I don't take many of them. 2 or 4, that's all. I can control myself. (…) I know this osteoarthritis of the knee will get worse. If I start taking medicine already, I think they won't work when I'll need them.” (Patient)*

The accommodations patients used to limit drug use were enduring pain, taking drugs during acute crisis or to prevent pain for special events that should not be spoiled by crisis, and reducing the dosage:

*“I am the one who knows when it hurts too much. If it is unbearable, I take painkillers. But (…) the painkiller I take gives me stomach problems although it really works on the pain. If I do feel pain but it's not too serious I take paracetamol. (…) I deal with it according to the pain. He (my doctor) gave me Diclofenac but it has never really worked” (Patient)*

Although the distinction between whether slow-acting symptomatic drugs for OA (SYSADOA) are drugs or dietary supplements is not absolutely clear in patients' minds, opinions were positive. Patients emphasize the positive effects on pain, the absence of significant side effects as compared with NSAIDs, and the importance of having these therapeutic agents when treatment options are limited. However, patients feel confident in abandoning these treatments without medical counseling when they consider their effects questionable. Dietary supplements are taken when prescribed by physicians or recommended by relatives:

*“A friend told me: “this year the doctor gave me cod-liver oil”. It's very important for the cartilage. We can also take shark cartilage as a dietary supplement. It's my GP who told me first that Harpagophytum was relevant for arthritis. And as they are all natural products, I thought “why not”?” (Patient)*

Dietary supplements are considered natural alternatives to pharmacological drugs. The image of “giving food” to joints is evoked:

*“I read cautiously all the things written on these products. And actually, when you have knee problems, it's as if the joint was not well-oiled. Dietary supplements feed the cartilage, and make the joints suppler. So it's getting better. We do have less pain (…) May be by getting older, the renewal of the cells works not so well. By taking these products, it does help my cells to renew.” (Patient)*

The absence of side effects or counter-indications is emphasized. To take dietary complements appears to be a compensative strategy in a context of few treatment options, which therefore corresponds to dissatisfaction with conventional drug therapy and constitutes auto-medication.

*Local treatments:* Local topical treatment is associated with the idea of pain relief and has a positive image. Local treatments are considered positively for different reasons: self-administration combined with massage is important for the mental construction of the image of pain relief;

*“Diclofenac cream helps me psychologically. It makes me feel better but it's psychological, I do know it doesn't really work. The cream is made especially for muscle pain, and in this case it is not that at all. The real thing is my cartilage is ruined, and the bones scrape together.” (Patient)*

Because local topical treatments are applied to the area below the administration site, local treatments match the strategy of “less drug therapy possible” sought by patients; patients' expectations seem to be lower for local treatments than for oral drugs, which might also decrease the risk of “being disappointed”.

Corticoid injection in the knee invokes ambivalent appreciations. Efficacy and rapidity of action are emphasized, but patients worry about the infiltration itself and the component injected, perceived as potentially weakening the cartilage. Hyaluronic acid injection in the knee is considered an alternative to surgery and drug therapy and has a positive image because it is thought to be a less aggressive procedure. Nevertheless, this treatment invokes extremely different appreciations, from totally ineffective to miraculously effective, concerning its efficacy.

*Non-pharmacological treatments:* Exercise therapy is considered essential after knee surgery to recover mobility and is important during the disease to increase muscle strength and relieve pain. Some patients regret the short-term effect of symptom relief, whereas others emphasize the lack of professionalism of physical therapists. Appreciations concerning spa therapy differ, some patients considering that it has substantial benefit and others considering it as a simple distraction. Knee orthosis is appreciated because of the reassurance given by the increased feeling of stability and because of pain relief attributed to heat. However, patients express aesthetic concerns and emphasize the burden of wearing an orthosis. Soles are considered complementary options to decrease weight bearing on the affected leg during gait. Assistive devices such as canes or wheelchairs are accepted as transitory options but are much less well accepted because they imply old age and loss of autonomy and because of the image reflected, if considered as permanent options.

*“This (wheeling chair) is awful! I don't accept it. I had to take it because I could not walk in my house (…) However, I don't want to go out with that. Maybe it is misplaced pride but it downgrades you. People stare at you and that annoys me. (…) because when we go for a walk or when we go shopping, and I no longer do that, people immediately look at you. I no longer do the food shopping. My husband does it.” (Patient)*

*“I don't want to meet people we know. For instance, I never go downtown with the cane. My husband does the food shopping. Neither I would go window shopping in Toulouse though I love that. First I get tired faster and I don't like people see me with the cane.” (Patient)*

*Interventions on the knee:* Joint lavage and arthroscopy are described as inducing only transient pain relief. Total knee arthroplasty is considered “the last-chance” medical procedure and is desired to occur as late as possible. Such therapy catalyzes general worries about surgical procedures – fears of anesthesia, nosocomial infections, failure concerning results – which can lead to increased disability. Patients express concerns about the lack of clarity concerning indications for surgery. The post-surgery period is also a cause for fear because it is perceived as long and painful. Patients who already experienced knee surgery have divergent assessments: some emphasize the recovery of functional performance and others express some deception about results.

*“I was a little disappointed by the first (knee surgery). The pain remained. I expected a better result. I've never been able to walk like I did before. I met people in physical therapy who were so happy with the surgery that they ran for the second knee. I have to say that I expected more than that…” (Patient)*

*Alternative therapies:* Patients cite several alternative therapies – acupuncture, osteopathy, homeopathy, naturopathy, phytotherapy, and Shiatsu – but express various opinions about the efficacy of these treatments. Reasons for choosing these therapies are to avoid long-term drug intake, delay the time for surgery, and their greater emphasis on prevention than biomedicine approaches. The use of dietary supplements gives the patients the feeling of being active, especially when confronted with fatalistic attitudes and trivialization of OA. Moreover, dietary supplements are not seen as a symptomatic or palliative answer but as a more satisfying option, an attempt to “cure the cause” of the illness. Reasons advanced for choosing or switching to alternative therapies are to have a physician directly administer the therapy and that physicians who deliver or prescribe alternative therapies be more accessible and open to discussion, have more empathy, spend more time with their patients, and consider patients more globally in their environment as compared with physicians prescribing biomedicine options.

*“At first, the acupuncturist asks me how I feel, and we talk too. I felt depressed occasionally, so it is another thing we can talk about. He can do something. He considers the patient as a whole, which is a real difference with physicians like the rheumatologist who examines you, asks you three questions and has finished with you. I do think the relationship with the doctor in alternative medicine is longer, deeper and makes more sense. I could tell you the same for the homeopath. With him it is at least 45 minutes; he asks many questions to have a global view.” (Patient)*

*Patients' self-implication in the treatment:* Patients develop additional strategies to decrease the effects of knee OA on pain and functional limitations such as the use of heat or cold. Some patients declare having modified their diet or doing exercise more frequently to better resist the effects of knee OA. These strategies are suggested by physicians or are the result of self-adaptation.

### Practitioner views

#### About the disease

Practitioners do not share homogenous perceptions of knee OA. These perceptions waver between a fatalist view of the disease with a trend to trivialization and a more voluntarist opinion emphasizing the consequences of knee OA on functional performance of patients and the need to modify the OA status in practitioners' representations. Practitioners' talks reveal a relative trivialization of OA in general and knee OA, which is perceived as the natural degradation of the body with age, a frequent and universal disease, and an ineluctable phenomenon (a fatality):

*“(Knee osteoarthritis) is part of getting older, it's normal really, not normal, but it makes sense as it is part of the natural evolution.” (GP)*

*“I will go further, I do not think it is a disease, it is a normal degeneration. It is inevitable. It is a more or less severe ageing depending on human beings. To me, it's more linked to ageing than to a disease, it's somehow inevitable. Everyone has osteoarthritis with ageing whereas not everybody has diabetes or hypertension… Or even cancer.” (GP)*

Representations of the seriousness of knee OA are ambivalent. Knee OA is considered potentially disabling:

*“It is a bloody nuisance, a thing which makes your life a misery and restricts your activities.” (GP)*

*“Knee osteoarthritis is a real barrier to a normal and nice life: they (patients) become dependent.” (GP)*

At the same time, its seriousness is weighed in terms of other diseases considered more serious:

*«And there are some more severe pathologies! With knee osteoarthritis, we don't have to provide the psychological support we have to do for cancer or diseases like that!” (Surgeon)*

Practitioners delivering alternative medicine have heterogeneous perceptions of knee OA largely depending on the type of care delivered. Knee OA causes are deducted from interpretation models of each alternative therapy (energetic disequilibrium for those delivering acupuncture, physiologic disequilibrium for osteopath, emotional disequilibrium for those delivering herbs):

*“I have a general answer one could say: it is an energetic deterioration; it is true that when you take the pulse, we almost find the same thing in everyone. That means that, at one stage, there is an energy that does no longer go through the energetic circuit, that damage the cartilage; then, it causes arthritis and we can feel it through the pulse. That's why I said it is a general answer.” (Acupuncture therapist)*

*“The interesting point is to know why this knee osteoarthritis appeared. It represents emotional worries that joy, rest and happy moments have not succeed to evacuate. Too much bad news, tough life experiences have for consequence that one day, you cannot deal with them anymore and keep them inside yourself. As a result, the body has to react and does it through out diseases and accidents. People have to investigate and be introspective. It may have several origins on a physiological aspect. But on the behavioural aspect, Bach Flowers are the one who will work. This may be due to overwork.” (Flower counselor and naturopathic practitioner)*

#### About the diagnosis

Practitioners (mainly GPs) consider that the aim of the first visit for knee symptoms is to distinguish between mechanical and inflammatory pain, which is sufficient to establish an appropriate prescription. The precise diagnosis of knee OA during this first visit is not considered crucial because it is not necessary for prescribing. Knee radiographs are considered essential for diagnosis confirmation and are prescribed after the first visit by practitioners who consider it important to have an early diagnosis or after the first line of treatment failed by those considering that early diagnosis is not crucial, generally before referring to a specialist:

*“At the beginning, we don't need to know for sure that it is knee osteoarthritis. If the pain calms down naturally, it means we have time to deal with it. If it is osteoarthritis, even if it is a rheumatic disease, it will become chronic and therefore come back from time to time.” (GP)*

As compared with practitioners delivering biomedicine, those delivering alternative medicine consider knee OA easy to diagnose and understand. Care providers who are not medical doctors tend to perform a more global (whole body) examination than those who are medical doctors, mainly to define causes of the disease and elaborate the therapeutic program.

#### About treatments and prescription strategies

Practitioners' representations of management steps are schematic and built according to flare-up treatments. The first step consists mainly of symptomatic pharmacological treatments and management by GPs; the second step consists in joint injections (mainly corticoids) and is managed by a knee specialist (rheumatologist in France); and the third step is joint replacement by the orthopedic surgeon.

*“First are anti-inflammatory drugs and then, depending on the evolution, if it develops slowly or not, we will go to injections. And the prosthetic knee is really the last option considered.” (GP)*

Prescription steps being limited, practitioners tend to optimize prescriptions by several strategies, varying the drugs within a same family, going back to the simplest prescriptions when they have not been previously prescribed, and associating complementary therapy (mainly between flare-ups with a prevention goal).

*“If (the patient) tells you “your last treatment did not work”, then you change. In the same therapeutic class, we try to select a more powerful drug, a little more effective.” (Surgeon)*

*“We realize that people with arthritis immediately take anti-inflammatory treatment. They do not even have Doliprane® where as, sometimes, just a half dose of it is enough. We have to explain that it is better for them to take Doliprane®, that it is less toxic than other treatments (Rheumatologist)*

Practitioners mentioned various treatments for knee OA and classified them in 3 general categories: flare-up treatments (NSAIDs, opioids, coticoids injections, joint lavage, alternative medicine), between–flare-up treatments (SYSADOAs, hyaluronic acid injections, physical therapy, knee braces, soles), and surgical treatments (osteotomy, total knee replacement).

Practitioners' opinions of the efficacy of drugs differ by the drug superfamily: analgesics are considered symptomatic treatments with limited effects, NSAIDs symptomatic treatments with frequent and serious side effects and numerous counter-indications, and SYSADOAs treatments without scientific proof of efficacy but with few and minor side effects that could help reduce other symptomatic treatments. GPs and rheumatologists consider total knee replacement the ultimate and only really efficient treatment, whereas orthopedic surgeons consider it one of the treatment options for knee OA. Care providers delivering alternative therapy have enthusiastic perceptions of the efficacy of the type of care they deliver. They are more critical of pharmacological treatment defined as chemica because they have side effects, inhibit cartilage regeneration and may be dangerous by hiding the symptom (pain), which is a useful yellow flag to know when knee should be put at rest:

*“(Practitioners) use chemicals and it compromises all our treatments: it pollutes them. They cure people with chemicals that get them crazy and dependent when natural products are available. They offer patients inefficient chemicals that might poison them and cause them diseases.” (Acupuncture therapist)*

*“Painkillers may also have another side effect: as the pain is being stopped, the patient will keep on his activities. He will play rugby and so worsen osteoarthritis. Pain is an alarm that has to be listened to. Killing the pain is not enough for the disease to disappear.” (Homeopath, acupuncture therapist and GP)*

#### The logics underlying the elaboration of prescriptions

The decision and elaboration of prescriptions for knee OA is a complex procedure (designated as “therapeutic do-it yourself”) combining several modalities of adaptation mobilizing medical and “common” knowledge. Prescription is fashioned by 3 logics: medical knowledge of the practitioner, the practitioner's representation of the treatments and the role they assign to each of these treatments, and the perception that the practitioner has of the patient and his/her expectations.

When practitioners elaborate a prescription, their objectives are not restricted to solving knee OA symptoms but are also to provide a response to patients' demands and expectations, optimize compliance to treatments, and minimize risks, including their own risks (mainly liability). According to their perceptions of treatments, practitioners can be differentiated by 4 attitudes determining therapeutic choices:

- those with a positive perception of existing treatments and consider that they can treat knee OA;
*“Now we get to put grafts such as for hair. There are hair grafts and we now manage to have cartilage grafts. We put small pads of cartilage that eventually over time spread like grass.” (Surgeon)*
- those with a pragmatic analysis of treatment and use available treatments;
- those who consider existing treatments insufficiently efficient and try to compensate in other ways, including organizing social activities for their patients to prevent against isolation and depression, regularly calling their patients, or developing a partnership with domestic help; and
- those who do not know how to deal with treatment options.

*“We, patients and physicians, are in the dark! (…) We are not comfortable with this pathology. (…) We really ought to know what are the impacts and the procedures according to the patients' profiles and risks.” (GP)*

Practitioners also take into account their patients' profile to adapt their prescription. Patients are classified according to 4 main variables: social characteristics, medical presentation, psychological profile, and activities. These variables lead to a very complex categorization of patient profiles; the tendency is to simplify to 2 main profiles determining therapeutic priorities: active (including sports), young patients, to whom surgery (conservative or not) should be proposed quickly, along with psychological support; and older, inactive patients with co-morbidities, to whom total knee replacement should be proposed as late as possible, for whom pain levels should be controlled and acceptable, and co-morbidities not worsened by pharmacological treatments. Implicitly, practitioners also classify patients in 2 categories: the “easy” patients, defined as living in the country, “not too old” with mild knee OA or very old resigned and fatalist; and the “complex” patients, defined as living in town, involved in sports, and those with obesity, co-morbidities, psychological distress, or professional claims. Medical doctors delivering alternative medicine tend to deliver biomedicine first and then alternative therapy when they define themselves mainly as a practitioners; those defining themselves as mainly care providers usually begin with alternative therapy. Those delivering alternative medicine more often emphasize diets and may forbid certain types of food.

#### Practitioners' expectations

Spontaneously, practitioners express few expectations concerning knee OA management. Nevertheless, analysis of interviews led to the identification of expectations concerning pharmacological treatments, outcome measures, prevention, medical education, and research. Concerning pharmacological treatments, practitioners expect treatment with structural efficacy that could slow or stop knee OA evolution:

*“We are all dreaming of a product which would rebuild the cartilage just by injecting it into the joints.” (GP)*

They also emphasize the need for medications with fewer side effects and counter-indications in order to increase therapeutic options. They also expect tools (decision trees) to help in therapeutic decision-making by defining treatments according to patient profiles:

*“We should have a decision checklist based on assessment of risks that would help us to identify how to proceed in 3 or 4 steps with knee osteoarthritis patients. We mainly need medical information. (…) We really need to know what are impacts on patients, the process to follow and the risks according to patients' typology.” (GP)*

Practitioners have reserved opinions about existing assessment tools mainly because they question their applicability in routine practice. However, some express expectations concerning tools better assessing certain dimensions such as psychological impact, personal life, and aesthetic burden. Practitioners emphasize that prevention could lead to earlier management of the disease at an earlier stage with probably more effective treatment strategies delivered to patients easier to treat because of fewer co-morbidities. They also express the need to focus prevention on the population at risk (the risk cited being professional status and overweight). Practitioners also express expectations about specific education and information focusing on the disease and its treatments, the stake again being help in therapeutic decision-making:

*“We should have more information, as practitioners, to know what to do. (…) I feel it is vague for us, and also for the patients! Practitioners of my age are not so confident with surgery… it (knee OA) is a pathology that makes us feel uncomfortable. There is no problem with hips surgery but when the knee is concerned, it is a frightening surgery.” (GP)*

Some practitioners deplore the lack of research interest in knee OA and others think it is not a priority:

*“There are very little studies done (…) there might be small teams of researchers working on osteoarthritis in various countries but there are no significant funds allocated to osteoarthritis. I recognize that if we want people to work till they are 70, we will need to do something against osteoarthritis!” (Rheumatologist)*

#### Patients' expectations seen by practitioners

Practitioners tend to differentiate 2 patients profiles: young, active patients with many expectations and old (or very old), resigned patients who consider knee OA a normal aging process, have few expectations and no longer believe in the efficacy of medicine. Practitioners consider that patients have expectations concerning symptoms (mainly pain and disability), visits to the practitioner (to be examined with special focus on auscultation and arterial tension recording and receive information on the diagnosis, prognosis and counseling), and prescriptions (demand for X-rays, propose drugs that are not over-the-counter, topics, recommendations concerning aesthetic burden for women). Practitioners identify specific but various patients' expectations concerning surgery (patients wishing to undergo surgery as soon as possible and others wishing to avoid this treatment option). They also have concerns about patients' unrealistic expectations about surgery such as returning to the functional performance lost a long time ago:

*“There are the objectively unsatisfied patients: when there is a problem with the prosthesis, when it is loosening or when there is an infection, but that scarcely happens. And there are the subjectively unsatisfied: the ones who always have pain; who no longer can walk three hours hunting; who are never happy with anything and who have thought that it (surgery) would bring them back 10 years before. The typical example is the 70 years old grand-mother with osteoarthritis everywhere and a rotten spine – sorry for the word but that's how we talk between us –who does not understand why she has not recovered and be as before.” (Surgeon)*

## Discussion

To our knowledge, this is the largest qualitative study of patients' and care providers' views of knee OA management in terms of number of patients and care providers interviewed and broadness of topics tackled.

### Patient views

#### Patient–physician relationship

The ideal patient–physician relationship is characterized by its flexibility, and satisfaction cannot be considered a simple accumulation of factors. The principle of physicians adjusting their behavior and practice to the patient seems to constitute the pivotal stake in satisfaction. Practitioners giving satisfaction should be both in the fields of consumerism and technical, social and moral competencies and are finally “summoned” to accept a perpetual adaptation process to changing states and profiles of their patients.

Differences may exist between patients' declared and real expectations. Patient often express expectations about information, for instance. Nevertheless, this expectation is variable, not uniformly shared between patients or during the therapeutic course. Moreover, this expectation can be less a need for more information than a need for re-assurance. The existence, formalization, accessibility and possibility of multiple returns for information probably matters more than a simple systematization and standardization of information.

#### Treatments

The patients' relationship with treatments, particularly drug therapies, is modulated by their own and others' experience with these treatments and by living with knee OA. It evolves over time with self-medication and self-modulation of dosages and variable degrees of compliance with prescribed therapies, sometimes leading to their being abandoned. Patients modulate prescribed medications according to 2 main criteria: relief of pain and physical or functional limitations and experienced or perceived risks or side effects. Patients with a long duration of knee OA seem to have these modulations.

#### Facilitators of and barriers to improving knee OA management concerning treatments

Patients' expectations regarding treatments vary according to the consideration of knee OA as an occasional or permanent problem. However, we identified a common attitude of resistance to change induced by this clinical situation, which suggests coping strategies similar to those observed for other chronic pain conditions such as low back pain [23].

All patients share the need for efficient symptomatic treatment strategies, but those considering knee OA as chronic emphasize the curative dimension of treatments and focus on more attention paid to causes and repercussions of knee OA.

Patients expect a shift in the management of knee OA from a technical viewpoint, centred on physical symptoms, to a more global viewpoint centred on the patient in all his/her dimensions. The stake is to promote knee OA management strategies that will not be limited to physical symptoms but will take into consideration the impact of knee OA on symbolic, temporal, relational, psychological, emotional, material, and physical dimensions. Patients emphasize the strategic importance of the patient–physician relationship in their satisfaction with knee OA management, the necessary flexibility of this relationship, and the risk of the “routinization” of management in chronic clinical situations being an obstacle to the adaptation of this management to the specificities of the patient's profile.

Dealing more accurately with patients' paradoxal representation of drug therapy is also a way to improve knee OA management. This issue raises the question of the conditions of optimizing drug therapy prescriptions. The equation to resolve combines 4 main dimensions: patients' representation of drugs (chemical, aggressive, harmful), their representation of the efficacy of drugs (material and symbolic), the more or less acceptable impact of the disease combined with the immediate demand of patients, and the possible contradiction between patients' way of life (routines) and the specificities (side effects) of prescribed dosages.

#### Practitioner views

Practitioners' views differ largely from patients' views, and the perception of patients' expectations by practitioners differs from those directly expressed by patients. This finding has already suggested in previous works in the field of osteoarticular chronic diseases [7]–[9].

Recent medical and social evolutions have led practitioners to manage patients in a context of relative uncertainty. These uncertainties influence how the medical visit is conducted, how professional time is managed and the level of practitioners' remuneration. In this context, specific to knee OA, the practitioner must be confident in the patient talk about symptoms, assessment of treatment efficacy, and compliance with treatment and counselling. Pain and disability are subjective symptoms difficult to assess, the difficulty being increased by the unwillingness of practitioners to use specific assessment tools for these symptoms. Practitioners must elaborate their prescriptions on this uncertain basis. We identified practitioners with 2 opposite attitudes: those who adapt their prescriptions to patient complaints, and those who prescribe the same treatment whatever the intensity of the complaint. When prescribing, practitioners must deal with 3 constraints: the feeling, mainly for GPs, of having imperfect information on therapeutic options (a limitation of their medical knowledge); having to deal with patients who have access to information and who want to take an active part in therapeutic decisions; and, to increase the acceptability of their prescriptions and therefore increase compliance, the necessity to explain and justify their therapeutic choices, which is time-consuming when they have professional time constraints. The main facilitators for increasing the quality of knee OA management seem to partly contradict constraints related to professional practice conditions.

In conclusion, this qualitative study exploring the views of patients and care providers of knee OA management suggest several ways to improve the patient–practitioner relationship and the efficacy of treatment strategies, probably by increasing their acceptability and compliance. The main factors of improvement we identified are providing adapted, formalized information to patients, adopting more global assessement and therapeutic approaches, and dealing more accurately with patients' paradoxal representation of drug therapy. Finally, we confirm that patients' and practitioners' views of OA largely differ, and more attention should be paid to patients' views to increase treatment adherence.
